# Genotypic characterization of HIV-1 C variants in PBMCs and cervicovaginal cells

**DOI:** 10.1186/1471-2334-14-S3-E8

**Published:** 2014-05-27

**Authors:** Varsha M Prabhu, Varsha S Padwal, Shilpa M Velha, Atmaram H Bandivdekar

**Affiliations:** 1 Department of Biochemistry, National Institute for Research in Reproductive Health, Mumbai, India

## Background

HIV-1 is primarily a mucosal pathogen since more than 80% of infections occur through genital exposure. Vaginal intercourse, though an inefficient mode of transmission, contributes more new infections worldwide than any other route. Also, the female reproductive tract has been identified as a compartment that harbors variants distinct from blood. Present study aims to highlight viral compartmentalization between these compartments reflecting inadequate ART penetration.

## Methods

In this study blood and cervicovaginal swabs were collected from 8 female subjects. CD4 counts and viral loads were determined. Translated amino acid sequences of C2 v3 region of env gene of proviral HIV-1 C in PBMCs and genital cells were analyzed using N-Glycosite and Geno2pheno [Co receptor] 1.2 programs for presence of N linked glycosylation sites and co receptor preference, respectively.

## Results

Characterization of translated amino acid sequences of C2 v3 region of *env* gene of HIV-1 C shows variation in the number of N linked glycosylation (NLG) sites and uniform co receptor preference. Viral load varies in blood and genital secretions.

## Conclusion

Genotypic characterization of viral variants in blood and female reproductive tract can provide information regarding their association with sexual transmission of HIV. Difference in the number of NLG sites observed may influence the affinity for host cell co receptor. Discrepancies in viral load of blood and genital secretions suggest that ART may not be uniformly effective in suppression of viral load in different compartments of the same individual.

